# Development of a Taxonomy of Policy Interventions for Integrating Nurse Practitioners into Health Systems

**DOI:** 10.34172/ijhpm.2023.8194

**Published:** 2024-01-22

**Authors:** Joshua Porat-Dahlerbruch, Shoshana Ratz, Moriah Ellen

**Affiliations:** ^1^Department of Acute and Tertiary Care, School of Nursing, University of Pittsburgh, Pittsburgh, PA, USA; ^2^Department of Health Policy and Management, Guilford Glazer Faculty of Business & Faculty of Health Sciences, Ben-Gurion University of the Negev, Be’er Sheva, Israel; ^3^Israel Implementation Science and Policy Engagement Centre, Ben-Gurion University of the Negev, Be’er Sheva, Israel; ^4^Institute of Health Policy Management and Evaluation, Dalla Lana School of Public Health, University of Toronto, Toronto, ON, Canada

**Keywords:** Advance Practice Nursing, Health Workforce, Implementation, Nursing, Nurse Practitioner, Nurse Practitioner Integration

## Abstract

**Background:**

Nurse practitioners (NPs)—nurses with advanced training who can provide and prescribe care—are increasingly prevalent internationally. The growth of NPs can be attributed to physician shortages, growing demand for health services, and the professionalization of nursing. Ensuring efficacious integration of NPs into the health system is critical for maximizing their impact on patient and system outcomes. Nonetheless, there is a dearth of information on practical policy interventions facilitating the successful integration of NPs. Effective policy interventions should capture the perspectives of actors at all levels of the health system—ie, national and organizational levels. This study aimed to delineate a taxonomy of policy interventions for integrating NPs into health systems. This paper presents a taxonomy developed among gerontology NPs in Israel.

**Methods:**

This qualitative descriptive study used multiple perspective, one-to-one interviews with four professional groups—national policy-makers, organizational administrators, NPs, and physicians. Data were analyzed using deductive content analysis. Analysis accounted for diverging and converging patterns between professional groups.

**Results:**

There were 58 participants across the four professional groups. The national-level domain interventions include marketing, workforce development, professional licensure and regulation, financial incentives, stakeholder cooperation, education and training programs, and national-level research. Organizational domain interventions included organizational guidelines, infrastructure development and resource allocation, interprofessional leadership engagement, and organizational messaging. Unit- and care-team domain interventions included interprofessional experience and exposure, team communication, and mentorship.

**Conclusion:**

The taxonomy’s trichotomy of three health system level domains describes the relationship between national and organizational policy interventions. Adopting these interventions may result in an improved response to provider shortages. Policies insufficiently addressing role clarity and workforce retention resulted in poor integration and a failure to efficaciously combat workforce shortages. Future work will expand the focus of this preliminary taxonomy by further development and testing with international participants.

## Background

**Key Messages**

**Implications for policy makers**

- These practical policy interventions can serve as a guide to policy development for integrating nurse practitioners (NPs) into the health system.
- The division of national-, organizational-, and care team-level interventions provides simple identification of the health system level for which the policy-maker is targeting.
- The taxonomy explains the relationship between national and organizational-level policy to enhance understanding of the effects of the policy interventions.
- These policy interventions for integrating NPs may facilitate an improved response to provider shortages.

**Implications for the public**

These findings will contribute to more efficacious integration of nurse practitioners (NPs) into the health system. As a result of adopting these policy interventions, NPs will likely be able to improve system-wide outcomes for patients. For instance, international research shows that when NPs can provide care efficaciously, access to care, appointment wait times, and patient satisfaction are improved. This taxonomy, furthermore, was developed to be broadly applicable across health system settings. The taxonomy targets policy interventions at government and organizational levels. As such, these study results can contribute to improved patient care and public access to care across the health system.

Nurse practitioners (NPs) are increasingly prominent internationally. An NP is a type of advanced practice nurse who “is educated to diagnose and treat conditions based on evidence-informed guidelines that include nursing principles that focus on treating the whole person rather than only the condition or disease.”^1^ While NPs are not the only type of advanced practice nurse who can prescribe, the level of practice autonomy, and accountability of NPs is determined by and sensitive to the country, context, and/or regulatory policies.^1^ There are a wide variety of role titles which meet the level of autonomy and multi-disciplinary practice, as defined by the International Council of Nurses.^2^ These role titles are often used interchangeably with the role title “NP.”^2^ For instance, in Ireland, there are advanced midwife practitioners, and in Israel, there are expert clinical nurses in a variety of disciplines, such as gerontology.^1,3^

Research in multiple nations has shown that NPs provide high care quality similar to physicians.^4-10^ NPs are especially well-suited to address health disparities as their training combines patient, family, and community aspects into care.^1,11-14^ This holistic approach to care is beneficial for meeting the growing demand for patient-centered care delivery.^1,15^

Introducing the NP role alone does not directly translate to improved system and patient outcomes. Improved outcomes are moderated by national and organizational policies facilitating efficacious integration of NPs into the health system.^12,14,16,17^ NP integration can be defined as “a multilevel process of incorporating NPs into the healthcare system to an extent at which they can function to the full extent of their education, training, and scope of practice and contribute to patient, health system, and population needs.”^16^ In several health systems internationally, empirical research and literature reviews have reported ongoing policy issues surrounding the integration of NPs.^8,18-23^ These publications attribute such policy issues to a dearth of knowledge of evidence-based policy interventions facilitating the efficacious integration of NPs.^2,20,24^

Most of the existing literature on NP integration has focused on identifying facilitators and barriers to efficacious integration.^16,17,22,25^ Understanding facilitators and barriers may be useful for identifying areas necessitating policy interventions to advance the integration of NPs.^2,26^ However, facilitators and barriers do not answer the pragmatic question, “What policies can be implemented to maximize the effects of facilitators and minimize those of barriers?”

Research has yet to develop a taxonomy of policy interventions facilitating the integration of NPs. A report from the Organization for Economic Cooperation and Development identified several broad policy tools promoting the integration of NPs in primary care.^17^ The identified policy tools included regular review of scope of practice laws/regulations, changes to financial and reimbursement schemes, and ongoing managerial support in organizations. Nevertheless, this report focuses on all advanced practice nurses and not necessarily NPs, who can prescribe and provide care and practice with high levels of autonomy. The policy tools, furthermore, are broad and lack practical guidance. Finally, the report focuses only on primary care and not on policies affecting care in other ambulatory nor acute care settings. Other reports of policy interventions for integrating NPs typically are embedded in the conclusions of studies conducted among clinicians in a specific setting.^27-29^

Recommended policy interventions in the literature, moreover, tend to lack a system-level perspective.^2^ Applicable policy interventions should account for all levels of policy.^30^ In NP integration, this includes national/jurisdictional policies (macro level), organizational structures (meso level), and individual clinics, units, or care teams (micro level).^31,32^ To capture the full range of contextual factors affecting NP integration, research should be conducted with participants who can speak to all three of these health system levels—ie, national policy-makers, organizational administrators, and clinicians.^2,16^

A growing body of literature demonstrates the generalizability of taxonomies guiding the implementation of evidence-based interventions, such as NP care.^2,30^ In other words, taxonomies can be designed for adaptability across multiple settings or countries.^30^ This holds true so long as a context component is incorporated into the taxonomy.^31,32^ High-level evidence, moreover, has demonstrated that the barriers and policy interventions for integrating NPs into health systems internationally is similar across countries.^6,16,17,25^ Together, these postulations render the development of a global taxonomy of policy interventions to guide NP integration feasible.^2^ This study aimed to delineate an applicable taxonomy of policy interventions at national and organizational levels for integrating NPs into the health system.

###  Policy Interventions for Gerontology Nurse Practitioner Integration in Israel

This reported study was conducted in Israel among national policy-makers, organizational administrators (eg, senior nurse managers), physicians, and NPs. The continuing work will expand the focus of the taxonomy through testing with an international audience. The integration of gerontology NPs, or NPs who care for older adults, was selected as the exemplar to develop this initial taxonomy because the role was introduced just ten years ago. Their ongoing integration reveals the success, outcomes, and needs of policy interventions for integrating NPs into the health system. Furthermore, gerontology NPs are authorized to work in nearly all care setting types—eg, ambulatory, primary, and acute care. Perspectives on gerontology NP integration, therefore, can capture integration issues across the nationwide health system.^23^

A regulatory framework exists for gerontology NPs in Israel, though constant revision and a priori policymaking have resulted in incomplete integration into the health system.^23^ Regulation and oversight for all NPs in Israel is centralized and occurs in the Ministry of Health’s Nursing Administration.^3^ The regulatory framework includes the role definition, scope of practice, licensure examination, and educational requirements.^3^ NPs can have a patient panel and practice autonomously aside from a required physician collaborative agreement. In Israel, gerontology NPs must possess a master’s, bachelor’s, and post-master’s certificate and work experience before admission to the program. The gerontology NP training program itself is a non-degree conferring program requiring 700-1240 hours.^3^ Ideally, this regulatory framework would address many integration issues. Recent research demonstrates, however, that several barriers to integrating the gerontology NP role in Israel exist.^23^ The study reports that many of these barriers in fact stem from an unclear regulatory framework and poor communication of the framework to the organizations which are integrating the NPs.^23^ As such, there is a need to delineate policy interventions to guide improvements in the regulatory framework and the integration of gerontology NPs in Israel, as a whole.

## Methods

###  Study Design

This study used a qualitative descriptive design to answer the research question: “What are policy interventions for integrating NPs into all levels of the health system?” Qualitative descriptive studies are appropriate when little is known about a topic and for recognizing the subjective nature of a topic with varying participant experiences.^33^ Qualitative descriptive approaches, moreover, are commonly used in health services research.^34^ Multiple perspective,^35^ one-to-one interviews with a semi-structured interview guide were used for data collection. There were four groups of participants, which together, provided a system-wide perspective that can speak to macro-, meso-, and micro-level policy interventions. In accordance with recommendations by Bradley et al,^34^ deductive content analysis^36^ was used. Initial policy intervention categories were synthesized. Converging, complementary, and diverging patterns between professional groups were identified using the guide by Vogl et al.^35^ Policy interventions were updated accordingly, and a final taxonomy of policy interventions across macro, meso, and micro health system domains was developed. We used Guba’s recommendations to ensure trustworthiness and rigor.^37^ Reporting of this study complies with the Standards for Reporting Qualitative Research (SRQR) (Supplementary file 1).^38^

###  Participants and Recruitment

This study used multiple perspective interviews^35^ in which viewpoints within the health system were collected from individuals with different professional roles—NPs, physicians, organizational administrators, and national policy-makers. A design involving multiple professional roles is critical for capturing a system-wide perspective accounting for macro-, meso-, and micro-policy levels. Multiple perspective interviews also facilitate a more comprehensive and nuanced understanding of the interactions between groups in a social system while still accounting for the life circumstances of individuals.^39^ Furthermore, much of the existing research addressing policy interventions for integrating NPs focuses only on clinicians (ie, NPs or physicians) in either uniform or lone settings (ie, only a single hospital or set of clinics).^2,16^ Indeed, all robust studies on policy interventions for integrating NPs are likely informative for policy intervention development. We posit, however, that studies not including policy decision-makers (ie, national policy-makers and organizational administrators) discount the needs and considerations of non-clinicians.^2^ Research should synthesize and compare the feedback of multiple participant groups—ie, NPs, physicians, organizational administrators, and policymakers—so that studies can be more applicable in policy development.^2^

This study focused on recruiting participants in Israel who worked specifically with gerontology NPs for several reasons. First, gerontology NPs in Israel work in nearly all care setting types—ie, ambulatory, primary, and acute care settings.^40^ Those who have worked with or delineated national policy for gerontology NPs, therefore, understand NP work in nearly all care setting types. Second, when this study was conducted, gerontology NPs were the largest clinical NP specialty in Israel with one of the longest histories of about 10 years. Finally, the needs of the older adult population are holistic, spanning medicine, mental health, and social care. The gerontology NP scope of practice embodies much of the unique, multidisciplinary approach to care provided by NPs.^6^

We aimed to recruit participants from a diverse set of care setting types across Israel to capture a nation-wide perspective. Recruiting a diverse set of participants enhances transferability and during analysis, their diverse perspectives can elucidate overlapping, complementary, and diverging themes.^30,41^ Recruiting from multiple work setting types and locations captures data reflecting professional relationships and societal nuances that may vary based on employer or region.^42^

 For inclusion in the study, NPs must have had at least six months of work experience. Physicians must have worked directly with gerontology NPs for at least six months. Organizational administrators must have been directly involved with organizational policy development for integrating gerontology NPs. Organizational administrators included a variety of upper-level managers, such as senior nurse managers, senior physician managers, and hospital chief executive officers. Finally, national policy-makers must have been involved with developing nationwide policies for NP integration. National policy-makers were defined as individuals who create or contribute to policy impacting the integration of NPs at a national level.

To recruit participants, we used purposive and snowball sampling.^43^ Participants recommended additional participants. The research team reviewed recommendations for fit with the inclusion criteria and potential of providing perspectives not yet captured in the data.^43^ Recruitment continued until saturation was reached within each of the four professional groups.^44^

 Participants were contacted via email, phone call, or text messaging for a maximum of three times at two-week intervals. If participants expressed interest in participating, a research team member set up a phone call with the participant to explain the study aims, voluntary participation, time commitment, and human subject research protections in place. Informed consent was received before beginning interviews. When necessary, the investigators or participants received organizational permissions to participate in the study.

###  Data Collection

Data were collected using a semi-structured, one-to-one interview guide. One-to-one interviews offer “deep” insight into social systems and lives while providing a space with freedom to express the participant’s views with privacy.^45^ Interviews were conducted in Hebrew or English depending on the participant’s preference. The interviews took place on Zoom, on the telephone, or in-person from November 2021 until August 2022. Questions asked participants to identify policy interventions to advance the integration of NPs in the macro, meso, and micro levels (Box 1). Participants were provided with descriptions of the terms “macro,” “meso,” and “micro.” Probing questions focused on clarifying participant recommendations and asking their thoughts about interventions commonly discussed by other participants. The interview guide was approved by all research team members. The guide was piloted by a sub-set of six participants and revised to ensure that the wording elicited responses addressing the study aim.^46^ Recordings were transcribed, de-identified, and stored on a secured server.

**Box 1. Interview Questions**

What policy changes would you recommend to improve the integration of gerontology NPs into the health system at the *macro* level?

What policy changes would you recommend to improve the integration of gerontology NPs into the health system at the *meso *level?

What policy changes would you recommend to improve the integration of gerontology NPs into the health system at the *micro *level?

In addition to these questions, probing and clarifying questions were asked. Participants were provided with the definitions of macro, meso, and micro health system levels.

-----------------

Abbreviation: NPs, nurse practitioners.

###  Data Analysis

The Guide for Qualitative Analysis in Health Services Research by Bradley et al^34^ directed the design of our analysis. The end goal was to develop a taxonomy. Taxonomies can be defined as a “formal system for classifying multifaceted, complex phenomena according to a set of common conceptual domains and dimensions.”^34^ Taxonomies are particularly useful for increasing clarity in defining complex phenomena.^34^ In this study, a taxonomy is useful for classifying and describing policy interventions at each of the health system domains—macro, meso, and micro.

Deductive content analysis was used, which is appropriate when the aim is to describe a phenomenon in conceptual form and the structure of analysis is operationalized on the basis of previous knowledge.^36,47^ Using the guide by Elo and Kyngäs,^36^ a deductive approach was used to create a coding structure—an initial code structure was developed before line-by-line review of the data.^48^ Prior policy interventions reported in some studies^17,49,50^ were used to create initial conceptual codes. Deductive coding with conceptual codes is widely used in developing taxonomies.^34^ Throughout analysis, the research team used peer debriefing to ensure that data were not forced into the pre-conceived codes.^51^ Instead, the process was iterative—the coding structure was revised as analysis progressed. Two researchers independently applied the finalized code structure to the data. After coding independently, the two coders met to compare. Discrepancies were discussed. Any unresolved discrepancies were discussed with a third researcher.^38^

With multiple perspective interviews, the research interest is typically at the group level, instead of the individual level.^52^ Focus is placed on the relationships between groups.^52^ For instance, certain interventions affecting costs may be more central for administrators and national policy-makers but less so for NPs and physicians. Team communication and collegiality may be a critical concern for NPs and physicians but may be less apparent to administrators and policy-makers. Contrasting a synthesized view of such perspectives, priorities, and nuances among participants within each professional group results in insights of relationship dynamics between health system actors.^41,53^ Importantly, contrasting group-level views enhances explanatory power more so than the sum of theories based on individual perspectives.^53^ An understanding of the dynamics, priorities, and interactions between clinicians and decision-makers elucidates interventions that will be well-received by and fit the needs of the major actors in NP integration policy development.^16,41^

Data were analyzed by the entire research team at the individual level and synthesized into categories of policy interventions with descriptions at the professional group level.^34,52^ Data were then triangulated (ie, participant triangulation)^41^ between the four professional groups—NPs, physicians, organizational administrators, and national policy-makers. Participant triangulation elucidates converging, diverging, and complementary patterns between participant groups.^41,52^ Converging, diverging, and complementary patterns for each policy intervention category were recorded and visualized in a matrix. Categories and their descriptions were revised based on the noted patterns—ie, new intervention categories were added, some were removed, and others were combined.^35^ This iterative re-categorization process continued until the research team was in agreement that the final list of intervention categories and corresponding descriptions reflected the data and nuances between professional groups. Interventions were organized according to macro, meso, and micro domains depending on which level the policy intervention would be enacted.^16^ The trichotomy of macro, meso, and micro levels was selected as it has been delineated previously in the published literature on NP integration.^2,16^

###  Trustworthiness

Several methods were employed to enhance trustworthiness according to the recommendations by Guba.^37^ First, we reflected on our personal, interpersonal, methodological, and contextual thoughts and potential biases during weekly research team meetings and writing personal memos.^54^ To enhance credibility, we used participant and investigator triangulation^41^ and provided direct quotes exemplifying the policy interventions.^55^ We verified accurate transcriptions and maintained an audit trail throughout analysis, which enhanced dependability.^55^ Peer debriefing facilitated confirmability.^56^ Transferability was enhanced by describing the methods and participant characteristics, which the audience can use to determine applicability of results to their setting.^55^

## Results

There were 58 participants in this study. Of all participants, 29% were NPs, 22% were physicians, 26% were organizational administrators, and 22% were national policy-makers. Of the 13 national policy-makers, five were Ministry of Health regulators, six were national health maintenance organization (HMO) decision-makers, and two were professional organization representatives. Over half of all participants had five or more years of experience in the role. Sixty-four percent of all participants were age 40-59. Each of the four professional groups contained participants from all regions in Israel—Tel Aviv metropolitan, Jerusalem metropolitan, and periphery regions. Fifty percent of participants worked in an inpatient hospital setting, 28% worked in an outpatient clinic, and 22% worked in a non-clinical setting. All participants working in non-clinical settings were national policy-makers. Table 1 displays the demographic characteristics of participants, categorized by professional group.

**Table 1:** Characteristics of Study Participants

	**NPs**	**Physicians**	**Organizational Administrators**	**National Policy-Makers**	**Overall Sample**
		**Overall Sample**			
Participants, No. (%)	17 (29)	13 (22)	15 (26)	13 (22)	58 (100)
		**Individual Characteristics**			
Experience in role, No. (%)					
Fewer than 2 years	8 (47)	2 (15)	3 (20)	1 (8)	14 (24)
2-5 years	5 (29)	1 (8)	3 (20)	3 (23)	12 (21)
5 or more years	4 (24)	10 (77)	9 (60)	9 (46)	32 (55)
Age, No. (%)					
20-39	3 (18)	3 (23)	2 (13)	0 (0)	8 (14)
40-59	13 (76)	5 (38)	10 (67)	9 (46)	37 (64)
60-79	1 (6)	5 (38)	3 (20)	4 (31)	13 (22)
		**Organizational Characteristics **			
Work region, No. (%)					
Tel Aviv metropolitan	2 (12)	4 (31)	6 (40)	6 (46)	19 (33)
Jerusalem metropolitan	12 (71)	8 (62)	7 (47)	5 (38)	31 (53)
Peripheries	3 (18)	1 (8)	2 (13)	2 (15)	8 (14)
Work setting, No. (%)					
Hospital					
Private or HMO	7 (41)	7 (54)	5 (33)	0 (0)	19 (33)
Government	2 (12)	2 (15)	6 (67)	0 (0)	10 (17)
Outpatient clinic	8 (47)	4 (31)	4 (27)	0 (0)	16 (28)
Non-clinical					
Ministry of Health	0 (0)	0 (0)	0 (0)	5 (38)	5 (9)
HMO	0 (0)	0 (0)	0 (0)	6 (46)	6 (10)
Professional stakeholders	0 (0)	0 (0)	0 (0)	2 (15)	2 (3)

Abbreviations: NPs, nurse practitioners; HMO, health maintenance organization. *Note:* Demographic data were collected during one-to-one interviews. Percentages describing characteristics were calculated by professional group.

 Figure illustrates the preliminary taxonomy. Each ring represents the three health system levels at which policy interventions would affect the integration of NPs—macro, meso, and micro. Within each ring, the taxonomy’s policy intervention categories are shown. The seven macro-level intervention groups are marketing, workforce development, professional licensure and regulation, financial incentives, stakeholder cooperation, education and training programs, and national-level research. The meso-level intervention categories are organizational guidelines, infrastructure development and resource allocation, interprofessional cooperation, and organizational messaging. Finally, the three micro-level categories are interprofessional experience and exposure, team communication, and mentorship.

**Figure:**
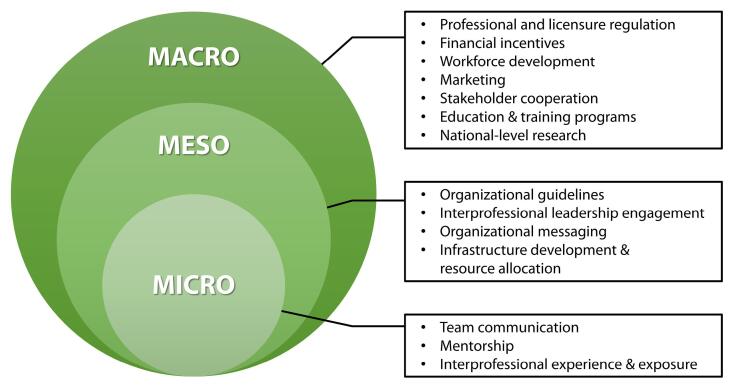
Illustration of the Preliminary Taxonomy of Policy Interventions to Integrating Nurse Practitioners into Health Systems. There are three domains in the taxonomy—macro, meso, and micro. Macro represents the national or jurisdictional level. Meso represents care organizations, such as hospitals and HMOs. Micro represents unit, individual clinic, or care team level. Descriptions of each policy intervention can be seen in Table 2.

Intervention descriptions were derived from the conceptual codes used to deduce policy intervention categories. The descriptions reflect the specific recommendations within each intervention category for improving the integration of NPs into the health system. In the text, we include these descriptions and direct quotes exemplifying policy intervention categories in the taxonomy. The descriptions also include explanations of topics in which the professional groups’ opinions converged and diverged. Summary descriptions for each policy intervention are provided in Table 2. Participants typically worded intervention recommendations as action items; the descriptions reflect this syntax.

**Table 2:** Preliminary Taxonomy of Policy Interventions for Integrating Nurse Practitioners into the Health System

**Policy Intervention**	**Description**
**Macro: Nation, Jurisdiction, Region**	
Professional and licensure regulation	Clearly define the NP role Delineate NP authorities and scope of practice Consider authorizing independent practice authority
Financial incentives	Reimburse NP care at a rate similar to physicians Provide financial incentives for organizations to hire NPs Incentivize physicians to supervise NPs Subsidize organizations to cover costs of sending nurses for NP education (ie, time off payments)
Stakeholder cooperation	Coordinate regulations and NP integration efforts with national-level physician and interprofessional groups Work with health professional, hospital, and HMO stakeholder groups when creating a national plan for integrating NPs *before* the introduction of the role Communicate NP prescriptive authority to pharmacies & home care certification authorities to social security offices
Workforce development	Increase the number of NPs trained and licensed Create and fund more government-allotted positions for NPs Establish NP salaries at a higher rate than “bedside” nurses
Education and training programs	Expand educational program content and rigor Consider additional clinical hour requirements after NP program completion Subsidize universities to open NP degree programs Establish NP program curricula based on existing international evidence
Marketing	Initiate an advertising campaign to inform the public of the new NP role Begin an information campaign on the NP role targeted at health professional colleagues Expose nursing school students to the NP role and career mobility
National-level research	Conduct national-level research on NP-linked patient outcomes Evaluate the current utilization of NPs across care settings
**Meso: Hospital, HMO**	
Organizational guidelines	Clearly define the NP role and authorities in the organization Specify the patients to be treated by NPs Create an organizational care model that includes NPs and recognizes prescriptive authorities
Infrastructure development & resource allocation	Increase salaries for NPs and clearly define pay schedules Employ NPs on most units or clinics, with priority given to units/clinics with the highest needs Place NPs in a wide variety of settings (ie, unit specialty, outpatient clinics, and home care) to widen exposure of patients and care team members Consider establishing a separate administration for NPs independent from medicine and nursing Establish a steering committee for integrating the NP role with representation from all organizational stakeholder groups. The committee should meet regularly before and throughout the integration process. Outline a formal plan for NP integration Systematically assess NP integration progress, communicate findings internally & update policies accordingly Give NPs access to prescribing and writing orders Allow patients to schedule primary care appointments with NPs Include role title, degrees, and certifications after NP names
Interprofessional leadership engagement	Host interprofessional workshops and seminars with physicians and NPs together Promote agreement and understanding of the NP role among organizational physician leaders Expose physicians and other health professionals to NP work before integrating them into a unit or clinic Administrators should meet regularly with physicians working with NPs to promote interprofessional cooperation with NPs Ask physicians to train the NPs with whom they will work
Organizational messaging	Communicate the NP role, education/training, and scope of practice *before* role introduction Clear supportive messaging from the central administration (both medical and nursing administrations) Allocate time to educate organizational leaders on NP care benefits
**Micro: Unit, Clinic, Care Team**	
Interprofessional experience and exposure	Demonstrate NP holistic care and value to the care team Introduce NPs and care team members *before* beginning full-time work Build gradual NP assimilation programs into care teams
Team communication	Institute regular care team meetings to promote consistent communication Educate NPs on interprofessional communication skills Educate patients and families about the NP role
Mentorship	Create mentorship relationships between senior and junior NPs and/or NPs and physicians

Abbreviations: NP, nurse practitioner; HMO, health maintenance organization. *Note*: Descriptions are based on conceptual codes. As such, they are worded as action items since this was the typical syntax used by participants

###  Macro

 Many of the participants emphasized that their colleagues and the public were unaware of the existence of the NP role. This hindered acceptance of NPs. As such, initiating a marketing campaign to inform the public and healthcare professionals of the NP role will enhance the understanding of the NP scope of practice and the willingness to work with or receive care from NPs. Marketing may also attract more nurses to the NP role and contribute to ongoing nursing professionalization.

“*The role needs to be advertised. At the national level, people do not know what an NP is at all. We alone can’t advertise the role—it has to come from the national level at the Ministry of Health so that people can know that there is such a thing as NPs” *~Policy-maker (HMO).

 Participants noted the need to invest in developing the NP profession. Particularly in nations like Israel with centralized health workforce planning, it is critical to increase the number of seats in NP programs and government-allotted positions for NPs. Otherwise, too few NPs will be trained, and NP workforce development will be hindered. Furthermore, the Ministry of Health should work toward approving additional NP positions in organizations which they have jurisdiction. Some NPs and administrators suggested requiring every organization to hire at least one NP.

 The NP role and scope of practice must be clearly defined at the national level. Organizational administrators and physicians emphasized that the lack of clarity rendered it difficult to develop a clear organizational NP role description and incorporate NPs into care models. Participants across all four groups also suggested that integration would have been more efficacious if greater emphasis had been placed on developing a clear role definition.

“*[Integration] depends on personalities…from one place it starts and goes and in others in starts and stops. We have examples of places with excellent integration processes…and in other places it has not moved much over time …[ these issues] arise because people do not have a clear idea of the NP role definition and authorities. They do not know how to employ them and incorporate them into the care team” *~Policy-maker (Ministry of Health).

 Financing schemes reimbursing NP care at rates identical or similar to physicians appropriately incentivize organizations to hire NPs. Lower reimbursement rates for NP care could result in a managerial preference to hire physicians over NPs—a point emphasized by administrators. Organizations may require financial incentives to hire NPs initially so that care team members can gain exposure to the role and understand their contribution to care.

 In the national planning process, involving interprofessional leaders is critical for downstream support in the field. Professional organization leaders and their communications highly influence the acceptability by clinicians in their respective profession. Some key interprofessional stakeholders included leaders from nursing, physician, and pharmaceutical professional organizations.

 NP training programs should be rigorous and prepare students for practice upon graduation. This is particularly critical because NP programs do not typically require post-graduation residency. The design of NP programs should meet international norms and standards. Current NP education in Israel occurs in non-university post-master’s certificate-conferring programs. A few physician participants noted the potential benefit of moving the program to a degree-granting university program, but the other three groups noted that this would add little benefit to the education quality. Policy-makers emphasized the added cost and bureaucratic complexity of moving all programs to a university setting. NPs noted that the certificate program along with the high pre-requisite standards (bachelor’s, master’s, and post-baccalaureate specialty certificate) have sufficiently prepared them didactically but not necessarily clinically.

 Nationwide research demonstrating the care quality and contribution of NPs may be critical for persuading care team members and organizational administrators to hire NPs. While policy-makers advocated for research, NPs, physicians, and administrators felt that it may have little effect on integration progress. Furthermore, it is critical to evaluate whether NPs are functioning as intended in the field and to identify facilitators/barriers to their integration requiring policy intervention.

“*For integration policies and practices, we need to speak from data and not from our senses of what is correct” *~NP.

###  Meso

 Professional leaders and administrators should define the NP role in care teams and models. Specific authorities and patient panels should be described, documented, and disseminated throughout the hospital. NPs and physicians highlighted the impact that unclear or non-existent organizational guidelines have on workflow and care team processes.

 Providing sufficient salaries higher than or equal to the salary of “bedside” nurses is critical for retaining the NP workforce. Many NPs described earning a lower salary as a result of working as an NP. Much of this, according to NPs and administrators, results from a limiting pay structure delineated by the Ministry of Health. Private hospitals, however, possess flexibility to determine salary structures independent of the Ministry of Health. Providing office space and provider-level electronic health record access is critical for function in an NP role.

“*I worked as the head nurse on my unit, and I earned a higher salary there than when I became an NP. It is not worth it for most people…even if they are earning more money as NPs, the salary increase is not worth it” *~NP.

Establishing an NP steering committee with the input of NPs and other key organizational leaders will facilitate the understanding of policies requiring revision to promote optimal NP practice. Similar to the intervention category *stakeholder cooperation*, the support of interprofessional leadership in organizations “trickles down”—a point emphasized by NPs and administrators.

“*The physicians are very critical. With the central organization administration at a hospital or a health maintenance organization, I would do a shared process with the medical and nursing administrations to manage the NP resource. In too many instances, the physicians are attracted to their side and the nurses to their side” *~Policy-maker (HMO).

 Clear supportive messaging from the organization’s administration is critical for promoting acceptance and support of NPs. Messaging can come in the form of electronic or verbal communications. Organizational administrators who emphasized positive and prolific messaging about the new NP role claimed that it had a widespread impact among all clinicians in their organization.

###  Micro

 All care team members should be exposed to NPs and understand their role in the care team.

 Time should be allocated before introducing NPs to educate clinicians about the NP role and the workforce’s potential benefits to patients. Physicians and NPs underscored that exposure to and experience working with NPs was the most critical factor impacting collegiality.

“*I recommend a conversation with a manager or administration with the medical nursing staffs to explain the [NP] function in the care team and what they are supposed to do…there should be a conversation before entering the care team” *~Physician.

 Regular interprofessional meetings inclusive of NPs are critical for establishing the NP role in the care team. Regular interprofessional communication fosters morale, according to NPs. It would also be beneficial to introduce patients and families to NPs and describe their education/training.

“*It will help the NPs to integrate if before they start working on the unit they have a conversation with the unit management, the physician staff, and the nursing staff to explain to them their functions and role” *~Physician.

 Establishing mentorship relationships between more senior and junior NPs or NPs and physicians facilitates collegiality. It can be useful for new-to-practice NPs to have a comfortable contact for questions and post-graduation transition-to-practice training.

## Discussion

 This study delineated a preliminary taxonomy of national and organizational policy interventions for integrating NPs into health systems. Recommendations were synthesized and compared between 58 participants from four professional groups—NPs, physicians, organizational administrators (eg, senior nurse managers), and national policy-makers. The resulting taxonomy consists of 14 policy intervention categories spanning three domains (macro, meso, and micro health system levels). Descriptions, grounded in the data, were provided for each of the policy interventions.

No research has delineated a taxonomy to guide policy development for integrating NPs. Prior international knowledge syntheses, nevertheless, have discussed policy interventions for integrating NPs into health systems.^6,17,57^ These studies are mainly based on literature reviews with additional conceptual work as opposed to this empirical study. Second, the studies tend to focus on nations with centralized health systems in the Organization for Economic Cooperation and Development. Finally, the policy recommendations are not synthesized into a practical taxonomy, which can be easily adoptable by policy-makers and organizational leadership. This taxonomy addresses these gaps in the science.

The taxonomy is intentionally broad as it can be adapted across multiple settings. Broadly applicable taxonomies for implementing interventions intended to improve healthcare delivery are becoming common with the increasing prevalence of implementation science in health services research.^2,30^ Furthermore, this taxonomy can contribute to the implementation science-based international NP Integration Model currently under development.^16^

The trichotomy between macro-, meso-, and micro-level policy dimensions is a key addition to the literature. The differentiation is critical for policy decision-making.^2^ The macro level targets national or jurisdictional level policymaking. These macro-level consumers include government and national professional organization policy-makers.^16^ The meso-level interventions target organizational decision-makers (eg, HMO or hospital system), such as senior medical or nursing management. Finally, the micro-level interventions target the working relationships within specific care settings, such as individual hospital units or clinics. While policies target specific decision-makers, the effects trickle down.^2^ In other words, effective macro-level policies must be in place for meso-level interventions to be successful. The same relationship exists between the meso and micro levels. The international empirical literature has indicated that such a hierarchical relationship exists.^16^ For example, a US-based study found that when controlling for state-level scope of practice restrictions, NPs working in poorer work environments (ie, insufficient resources and poor working relationships with colleagues) were less likely to report higher care quality.^58^ The key in this study is the necessity to statistically control for scope of practice restrictions because their effect on ability to provide high-quality care is independent from the effect of the work environment.^58,59^

One recurring theme emerging from the data is the need to address role clarity at all health system levels. This can be seen in the taxonomy—*professional and licensure regulation* (macro), *organizational guidelines* (meso), and *interprofessional experience and exposure* (micro). These findings are consistent with research across countries, which reported that poorly defined NP role definitions or scope of practice delineation at the macro level has downstream effects impacting the meso and micro levels.^7,9,10,17,21,23,27^ According to these studies, the lack of clarity resulted in inconsistent recognition of NPs by financial institutions, care organizations, and care team members. This study’s taxonomy suggests several specific recommendations for policy interventions to address these role definition and scope of practice issues (Table 2).

Another over-arching theme across macro and meso levels is the connection between financial incentives and workforce retention. These results suggest a need to develop policy reimbursing organizations for NP care at rates similar to physicians. Otherwise, care organizations have less incentive to hire NPs whose productivity and lower reimbursement rates may not offset their onboarding and employment costs. Furthermore, this study showed that nurses licensed as NPs have little incentive to work in NP roles if centralized workforce regulators do not approve NP compensation appropriately for their expanded role. This interaction of macro- and meso-level policies is not unique in Israel.^23^ International knowledge syntheses report similar findings across countries.^16,17,25^

A potential consequence of policies not addressing financial incentives or role clarity is a failure to address workforce shortages, a key impetus to introducing the NP role in many countries.^16^ In the Israeli context, research has demonstrated difficulty attracting nurses to the NP role when higher compensation is not guaranteed.^23^ Research has also shown that when organizations in centralized health systems are not provided with a sufficient number of NP positions, they will not hire or retain NPs.^17,23^ Finally, insufficient role clarity has been linked to poor NP-reported work environments, NP turnover, and lacking organizational accommodation for the NP role.^10,23,60^ Together, these financial and role clarity issues result in poor NP integration and an inability to fulfill one of the strongest impetuses, or antecedents, for introducing NPs into a system or organization—addressing provider shortages. Interventions from this taxonomy can be adopted to address the impact of financial and role clarity issues.

Nonetheless, this taxonomy likely requires further development to expand its generalizability to an international audience. Much of these generalizability limitations stem from the current state of Israel’s NP regulatory framework. Indeed, the framework defines scope of practice and role definitions. Clinicians and organizational administrators, however, reference its lack of clarity and inadequate knowledge of policies to facilitate the integration of NPs in practice.^23^ The NP education requirements, furthermore, are unorthodox—a post-master’s certificate program with a master’s degree and post-baccalaureate specialty certificate as pre-requisites for admission. Other countries do not have such NP education requirements nor is the model consistent with the International Council for Nurses’ recommendations.^1^ These integration issues, in spite of the presence of an NP regulatory framework, are not unique to Israel—research has reported similar integration delays in Singapore and France.^10,21^

 Future research should focus on reaching consensus among international experts. Experts can provide feedback on the relevance and international applicability of the policy interventions and the corresponding descriptions. Furthermore, this model of study accounting for multiple perspectives may be useful in studying the integration of any healthcare occupation into the health system.

###  Strengths and Limitations

 This study had several strengths. First, this study included participants from four professional groups representing a system-wide perspective on integrating NPs. Most research on the integration of NPs into the health systems includes only NP and/or physician participants. Excluding organizational administrators and national policy-makers limits the usefulness of results. Another strength is the implementation science lens applied to the study. Implementation science is unique in its methods enhancing the generalizability of taxonomies. The aim of this study, both in developing the preliminary taxonomy and in future development, is to create a global taxonomy of policy interventions for integrating NPs. Prior research delineating policy interventions for integrating NPs has not applied implementation science.

 This study has several limitations. First, the study was conducted in one country. Israel was selected as NPs are actively integrating into the health system, rendering it an exemplar for understanding effective policy interventions. However, the longer-term goal is to develop a global taxonomy. As such, further development is necessary to broaden the taxonomy’s international applicability. Second, the study was conducted with gerontology NPs and those who have worked with them or developed policy for their integration. Gerontology NPs were selected because they are trained and authorized to work in nearly all care settings (eg, inpatient hospitals, community, and long-term care settings). The gerontology NP breadth of training, scope of practice, and authorities led us to posit that focusing on this specialty would be most broadly applicable to other specialties. However, a global taxonomy must be applicable to nearly all NP specialties. As such, further international development is necessary before the taxonomy can be adopted among other specialties.

## Conclusion

 To improve the integration of NPs into health systems, national policy-makers and organizational administrators would benefit from a taxonomy of policy interventions. Adopting these interventions could result in an improved response to provider shortages—a key antecedent to NP integration. The taxonomy will likely be useful for advancing the integration of NPs outside of the Israeli context once further developed through additional research.

## Acknowledgements

 We would like to thank Ivy Chen and Branden Dutchess for their contributions to editing, formatting, and refining this article.

## Ethical issues

 This study was approved by the Ben-Gurion University Human Subjects Research Committee [ME14112021]. All participants provided written informed consent prior to study participation.

## Competing interests

 Authors declare that they have no competing interests.

## Supplementary files


Supplementary file 1. SRQR Checklist.
